# A keypoint-based method for detecting weed growth points in corn field environments

**DOI:** 10.1016/j.plaphe.2025.100072

**Published:** 2025-06-20

**Authors:** Mochen Liu, Xiaoli Xu, Tingdong Tian, Mingrui Shang, Zhanhua Song, Fuyang Tian, Yinfa Yan

**Affiliations:** a College of Mechanical and Electronic Engineering, Shandong Agricultural University, Tai'an, Shandong, 271018, China; b Shandong Higher Education Institution Future Industry Engineering Research Center of Intelligent Agricultural Robots, Tai'an, Shandong, 271018, China

**Keywords:** Weed detection, Keypoints, Corn seedlings, Growth points, Precision weeding

## Abstract

Weed growth significantly impacts corn yield. With the continuous development of weed control technologies, achieving more effective and precise weed management has become a major challenge in corn production. To achieve precise weed suppression, this study proposes a growth point detection method based on a keypoint pose estimation model capable of effectively detecting various weeds and locating various weed growth points during the 2nd–5th leaf stage of corn development. To address the complex working environment of precision weeding machines in corn fields, including occlusion, dense growth, and variable lighting conditions, we design a dilation-wise residual module (DWRM) for the detector and a separation and enhancement attention module (SEAM) for pose estimation to adapt to these challenges. Furthermore, owing to the limited computational resources in field settings, we introduced the RepViT block (RVB) to achieve model lightweighting. The proposed method was evaluated on the constructed corn field dataset. The experimental results demonstrated that SRD-YOLO achieved an ${mAP}_{kpt}$ of 96.5 ​%, an F1 score of 94 ​%, and an FPS of 169, while reducing the model parameters by 8.7M. SRD-YOLO effectively meets the requirements for growth point localization under challenging conditions, providing robust technical support for real-time and precise weed control in corn fields.

## 1 Introduction

Corn is a globally important food crop and serves as a source of raw materials for products such as poultry and livestock feed, as well as food additives [1]. Weed control has long been a persistent challenge in the agricultural sector [2]. The early developmental stage of corn, specifically the 2nd–5th leaf stage, is a critical period for the rapid growth of root systems and vegetative organs. At this time, corn seedlings are still small, and if weeds are not effectively controlled during this period, they will compete with corn for water, nutrients, and light, which can significantly affect the final yield and quality of the crop. Missing this window can lead to irreversible reductions in crop competitiveness and final yield due to weed interference [3]. Furthermore, after the 5-leaf stage, corn generally gains a competitive advantage over weeds, and frequent weed suppression efforts are no longer necessary.

To achieve sustainable agricultural development, weed control methods have gradually evolved toward intelligence, precision, and environmental friendliness [4], leading to more efficient and environmentally friendly solutions, such as precision spraying and laser weeding. These methods control weeds by precisely inhibiting their growth, and precise inhibition of weeds during the seedling stage of corn can enhance field management quality, improve efficiency, and promote eco-friendliness. The premise of precise weed inhibition technology is accurate identification of the growth points of weeds, specifically their apical meristem regions. However, the complexity of corn fields, which are characterized by diverse weed species, complex backgrounds, and variable lighting conditions, poses significant challenges for distinguishing crops from weeds and accurately locating weed growth points. With the gradual application of deep learning in agriculture, machine vision technology has been extensively studied and applied in weed segmentation and detection [5], providing a potential solution for identifying weed growth points in corn fields.

In the field of weed detection, deep learning algorithms based on machine vision detect plants as a whole through bounding boxes or segmentation, enabling the distinction between weeds and crops. Zou et al. [6] proposed a weed segmentation algorithm based on U-Net [7], which was trained by a two-stage training method composed of pretraining and fine-tuning, achieving accurate weed segmentation under complex field conditions. Kim & Park [8] introduced a multitask semantic segmentation-convolutional neural network (MTS-CNN) with single-stage training, which enhances the correlation between crop and weed categories. This approach resolves the issue that certain categories of crops or weeds are not detected correctly because of the large difference in segmentation performance between different crops and weeds. Chen et al. [9] designed the YOLO-sesame model on the basis of the YOLOv4 model, which successfully addresses the challenge of accurately detecting weeds in sesame fields. This model is particularly effective in cases where sesame seedlings and weeds share similar shapes and where the weeds vary greatly in size and characteristics. Liu et al. [10] proposed an improved YOLOv4-tiny model integrated with a multiscale retinex with color restoration (MSRCR) enhancement algorithm, which improves image contrast and detail quality, reduces model parameters and memory usage, and achieves high-precision real-time detection of corn seedlings and weeds in field environments. Fan et al. [11] presented a weed detection model based on Faster-RCNN that is capable of distinguishing weeds from cotton seedlings under complex growth conditions.

The aforementioned studies focused on segmentation and detection methods for weed recognition. These methods indicate the overall position of weeds but do not accurately identify their growth points. As a result, precision weeding techniques, such as laser weeding, may target weed branches and leaves, thereby reducing the effectiveness of weed suppression. Keypoint detection, initially applied in human pose estimation, has now been widely adopted in agricultural applications. Sun et al. [12] proposed a novel method for detecting keypoints of fruit branches, providing coordinates for branch pruning during fruit harvesting. Shuai et al. [13] introduced a method for detecting keypoints and picking positions of tea buds under complex environments, allowing precise localization of tea leaf picking points on the basis of keypoint information. Dun et al. [14] proposed a tomato posture detection algorithm (TPD) based on YOLOv5-lmk and a point cloud processing module, which detects tomato bounding boxes and calyx center keypoints, providing a theoretical foundation for 3D posture detection of tomatoes. Liu et al. [15] developed a method based on the improved YOLOv8-Pose [16] model for identifying ripe strawberries and detecting peduncle keypoints under greenhouse cultivation. This method effectively detects ripe strawberries and accurately predicts and marks peduncle keypoints. He et al. [17] proposed a bottom-up keypoint detection model, DEKR-SPrior, which enables more efficient localization and counting of pods in soybean.

To achieve precise inhibition of weeds in corn fields, this study proposes a keypoint-based method to identify and locate weed growth points. The main contributions of this study are as follows:

(1) The proposed method is based on the YOLOv8-Pose network, which treats growth points as keypoints and target bounding boxes as detection frames. It enables the classification of corn and weeds, as well as the localization of weed growth points. The backbone network is improved using the dilation-wise residual module (DWRM), which reduces the difficulty of extracting multiscale contextual information from growth points. This enhancement allows the model to capture more comprehensive features even under conditions of dense weed growth and high species diversity.
(2) Given the limited computational resources available in field environments, the C2f structure is optimized using the RepViT block (RVB). This modification reduces computational overhead and memory usage, thereby saving computational resources.
(3) Occlusion is a common issue during field operations and often affects operational efficiency. To address this, the separation and enhancement attention module (SEAM) is incorporated into the head structure, emphasizing weed regions in the images and compensating for occluded features in the original data.

## 2 Materials and methods

### 2.1 Date acquisition and expansion

Images of corn seedlings at the 2nd–5th leaf stage and their associated weeds were collected to construct a dataset. The collection time was divided into two phases: the first phase took place from September 18 to September 19, 2023, featuring the corn variety Denghai 11, whereas the second phase occurred from May 8 to May 9, 2024, featuring the corn variety Nongda 108. Considering that agricultural robots are designed to operate continuously across a 24-h cycle (including nighttime conditions), it is essential to account for variable lighting environments in both model training and evaluation. To simulate the operational context of agricultural robots better, we collected image data under different illumination scenarios. The daily collection time slots were from 12:00 to 13:00, 17:00 to 18:00, and 20:00 to 21:00. The three time periods correspond to conditions of strong daylight, weak daylight, and nighttime illumination. For nighttime image collection, we used LED lighting as the light source. The LED equipment used was the Sidande fill light (36 ​W, manufactured in Shenzhen, China). This diverse acquisition strategy enhances the model's robustness to varying lighting conditions and improves its generalizability in real-world applications. The images were captured in the corn experimental field at the Panhe Campus of Shandong Agricultural University (117.159251°E, 36.158780°N) using a Raspberry Pi Camera V2 (Raspberry Pi Foundation, China). This camera, equipped with a SONY IMX219 image sensor, has a maximum resolution of 3280 ​× ​2646. The camera was shot vertically 60 ​cm above the ground, and a total of 1736 images of the corn seedlings and their accompanying weeds were obtained while they were stored in PNG format. Among these images, 1048 field images were taken during the daytime, and 688 were taken at night.

**Fig. 1:**
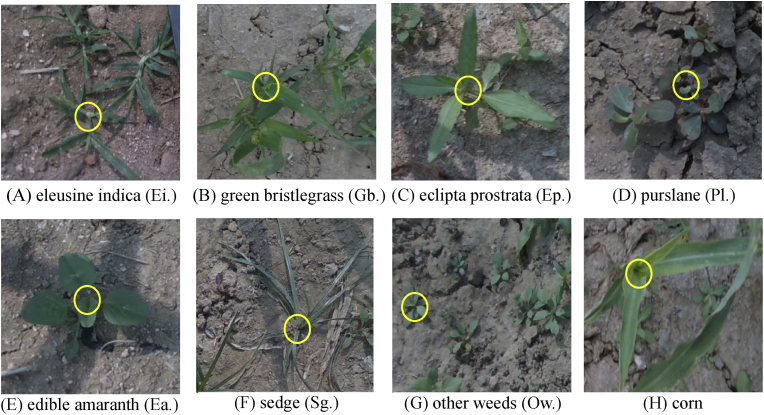
Images of corn and its associated weeds. Yellow circles are locations of growth points.

The companion weeds of corn in this dataset were classified into seven categories, namely, Eleusine indica (Ei.), green bristlegrass (Gb.), purslane (Pl.), edible amaranth (Ea.), sedge (Sg.), eclipta prostrata (Ep.), and other weeds (Ow.), which are representative of the typical corn-associated weed species in North China [[18], [19], [20]]. The dataset includes eight labeled categories, including corn, as shown in Fig. 1. Images (A), (D), and (F) demonstrate that the growth points of Ei., Pl. and Sg. are located at the roots, respectively. Notably, Pl. exhibits an irregular shape, with cotyledons that are compact and overlapping. Moreover, (A), (C), and (E) show that the growth points of Gb., Ep. and Ea. are located on the stem. The growth points of Gb. and Ep. are positioned directly at the apex of the stem, whereas Ea. exhibits diamond-shaped ovate leaves that grow in a dispersed pattern, with the growth points located at the upper end of the stem where branching is dispersed.

The dataset was manually annotated using the image annotation tool Labelme. Considering the need to avoid damaging corn seedlings during the actual weeding process, the labels were divided into corn and its seven associated weed species. During annotation, the growth points of the targets were marked as keypoints, while the minimum bounding rectangles of the targets were used as detection boxes. The annotations were stored in JSON file format. To enhance the network's generalization ability, data augmentation techniques such as translation, horizontal flipping, and brightness adjustment were applied to the original images, expanding the dataset to 3380 images, comprising a total of 49,295 labels. The label classification information is shown in Table 1. The augmented dataset was randomly divided into training, validation, and testing sets at a ratio of 7:2:1, resulting in 2366 images for training, 676 for validation, and 338 for testing.

**Table 1:** Label information of the dataset.

Label classification	Number of labels	
	Daylight	Night
Ei.	3292	2243
Gb.	1839	1361
Ep.	5370	3765
Pl.	1443	1087
Ea.	1887	1103
Sg.	1319	1126
Ow.	9901	8514
Corn	3021	2024

The images in the dataset depict various growth states of weeds, including weeds coexisting with corn seedlings, dense weed distribution, and sparse weed distribution. This dataset presents the following detection challenges: weed growth points are obscured. A large variety of weed species are present, with uneven distributions. Varying light intensities are present (nighttime images were captured using LED lighting), with strong light intensities producing significant shadows, and the growth point features of weeds are less distinguishable under nighttime lighting. Field obstructions such as irrigation pipes can obscure weeds. These challenges are illustrated in Fig. S1.

### 2.2 Construction of the SRD-YOLO detection model

#### 2.2.1 SRD-YOLO

To achieve accurate weed growth point detection and realize lightweight deployment, this study is based on YOLOv8-Pose for model construction. YOLOv8 is a detection algorithm in the YOLO series [[21], [22], [23], [24]] that is designed for tasks such as object detection, image classification, keypoint detection, and instance segmentation. In this study, YOLOv8s, a smaller-scale model that meets accuracy requirements, was selected as the base model. The network structure of YOLOv8s consists of three main components: the backbone, neck, and head.

For the pose module, the YOLOv8-Pose network draws inspiration from YOLO-Pose [25], a method for human pose estimation. On the basis of the YOLOv8 network, a pose branch is added to the detection head to perform keypoint detection tasks. The detection head outputs three types of information—cls, boxes, and keypoints—for each grid cell, enabling predictions of the target's category, anchor box, and corresponding keypoints. Each keypoint includes its position and confidence level, represented as x, y, and conf. The confidence score (conf) represents the model's certainty regarding the visibility or presence of a keypoint within an object instance. It is directly predicted by the network and expressed as the output of a sigmoid activation function. Thus, the pose detection branch associates N keypoints with each anchor point, resulting in N ​× ​3 elements.

For pose detection with n keypoints, the prediction vector $P_{v}$ at each anchor is defined as:

(1) $$P_{v}=K_{x}^{1},K_{y}^{1},K_{conf}^{1},\ldots ,\ldots ,\ldots ,K_{x}^{n},K_{y}^{n},K_{conf}^{n}$$

**Fig. 2:**
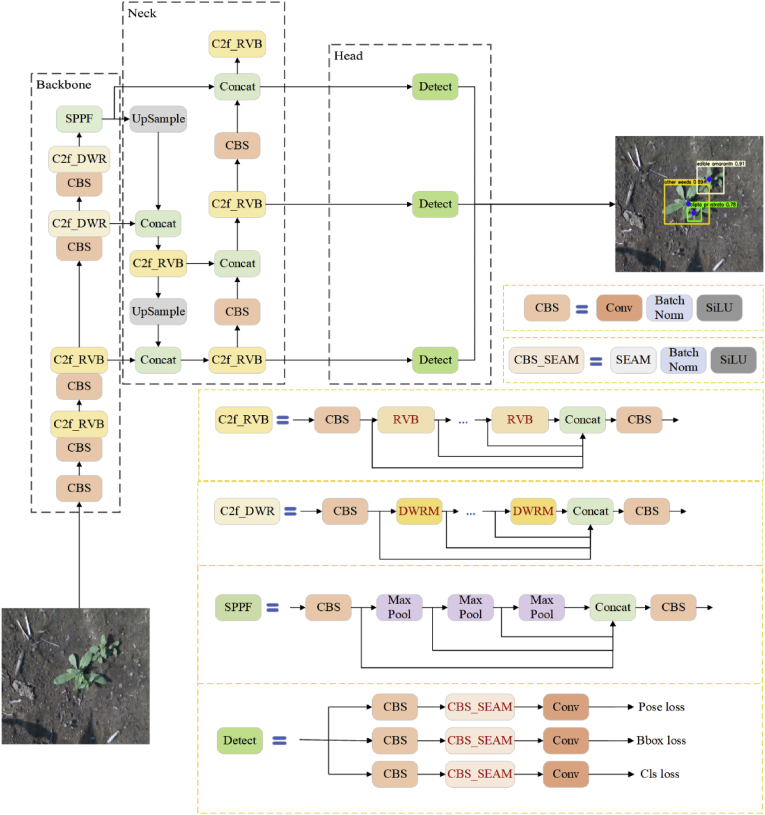
Architecture of the SRD-YOLO network. The parts in red font represent improved modules.

The construction process is as follows: In the backbone network, the DWRM is incorporated to improve the C2f structure in the higher-level convolution layers, enhancing the model's ability to extract multiscale contextual information of targets. This enables effective weed classification and growth point detection under conditions of dense growth and varying light intensities. For the lower-level convolution and neck portions, the block module of RepViT was utilized to optimize the C2f structure, achieving lightweight deployment. Finally, SEAM was integrated into the detection head to improve the performance of the pose branch in detecting keypoints under occlusion. The improved model is named SRD-YOLO, and its architecture is shown in Fig. 2. The specific details of the improvements will be further elaborated.

#### 2.2.2 Dilation-wise residual module

Field weeds exhibit a wide variety of species and uneven distributions, often resulting in clustered weeds and densely packed weed growth points, which lead to missed detections during growth point identification. Additionally, in nighttime images or daytime images with shadows, the features of weed growth points are less distinct, making keypoint detection significantly more challenging than in shadow-free daytime images. Therefore, the detection of weed growth points requires the establishment of long-distance semantic connections to extract growth point features effectively. In this study, a dilation-wise residual module (DWRM) was introduced into the high-level convolution layers of the backbone of the YOLOv8-Pose model. This enhancement improves the model's ability to capture the multiscale contextual information of targets [26].

**Fig. 3:**
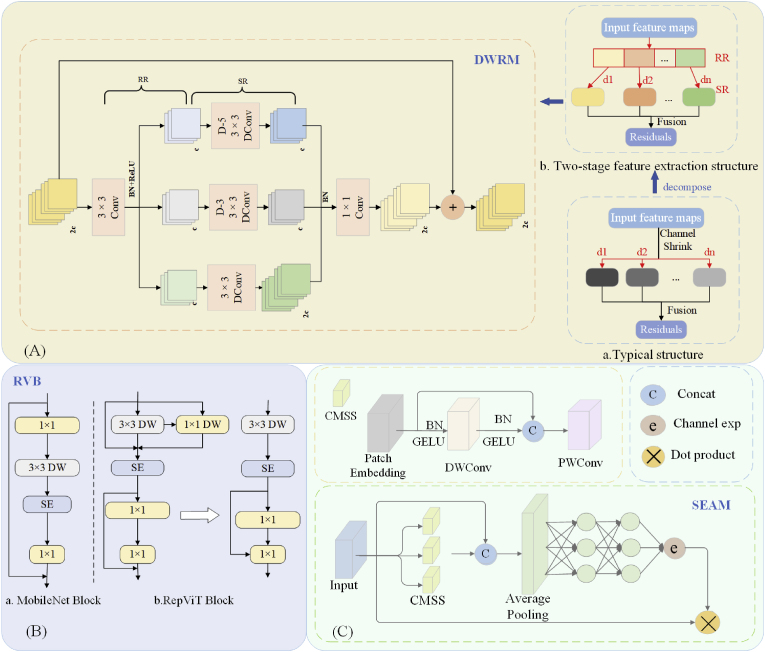
Structural diagrams of the three modules. (A) Schematic structure of the DWRM, where dx is the expansion rate, x ϵ (1,n), D-n denotes extended convolution with an expansion rate of n, + in the circle denotes the addition operation, and c denotes the base of the feature mapping channel. (B) The block design of RepViT. (C) Illustration of SEAM. The bottom is the architecture of the SEAM.

The DWRM is a module designed according to structure (b) in Fig. 3(A), while structure (b) is decomposed from (a). The structure of (a) is a typical structure for extracting the target multiscale context information, and feature extraction is directly performed by multi-rate depth-extended convolution. To reduce the difficulty of extracting the target context information, this process is decomposed into two steps to obtain the structure of (b).

The structure of the DWRM is shown in Fig. 3(A), which also performs context extraction in two steps. The first step is region residualization (RR), which extracts the initial features by a 3 ​× ​3 ordinary convolution and activates the region features by combining the batch normalization (BN) layer and the ReLU activation function. The second step is semantic residualization (SR), in which the regional features are first divided into several groups, with each group processed by dilated convolutions at different rates. This approach allows the first step to learn the required features on the basis of the receptive field size determined in the second step, achieving reverse matching of receptive fields. This method effectively captures multiscale contextual information of the target. The outputs from multiple groups are subsequently aggregated, normalized through a BN layer, and merged using pointwise convolution to form the final residual. Finally, the final residual is fused with the input feature map to obtain more comprehensive features.

#### 2.2.3 RepViT block

Precision weeding robots operate in the field with limited device computational resources. To facilitate the deployment of models on mobile devices, this study introduces the RepViT block (RVB) to enhance C2f in the backbone and neck of the YOLOv8-Pose model to achieve a balance between lightweight and high accuracy [27].

The RVB architecture emulates the existing lightweight ViTs by splitting the token mixer and channel mixer in MobileNetV3-L. The block portion of MobileNetV3 is shown as (a) in Fig. 3(B) [28], which implements interactions between channels through the 1 ​× ​1 expansion layer in the first stage and the 1 ​× ​1 projection layer in the fourth stage. (i.e., channel mixer). The 3 ​× ​3 depthwise (DW) convolution layer in the second stage is then used for the fusion of spatial information (i.e., token mixer). This design couples the token mixer and the channel mixer together. The block structure of RepViT is shown in (b), which moves the DW layer and the squeeze-and-excitation (SE) layer to the front, separating the token mixer and channel mixer using structural reparameterization. Thus, some of the associated computational and memory costs in the inference process are eliminated. Additionally, RepViT decreases the expansion ratio while increasing the network width, thereby saving computational resources and reducing the overall inference time.

#### 2.2.4 Separation and enhancement attention module

In the natural field environment, corn and weeds have mutual occlusion, and when the growth point of a weed is occluded, it has a certain effect on the detection accuracy of the growth point. To improve the detection accuracy of the growth point in the case of occlusion, this study introduces the separation and enhancement attention module (SEAM) in the head part of the YOLOv8-Pose model to compensate for the occluded features [29] and improve the detection accuracy of the pose branch for the keypoints. Its structure is shown in Fig. 3(C).

SEAM is a multi-head attention module that leverages the relationships between feature mappings to enhance occluded features. This approach serves two primary purposes: emphasizing regions of weeds in images and compensating for the occluded features in the original feature map. Its architecture is depicted in Fig. 3(C). The input first passes through the channel and spatial mixing module (CSMM), which serves as a combined module for spatial and channel interactions. The output of the CSMM module undergoes average pooling for channel expansion. Finally, the output of the SEAM is multiplied with the original feature map as attention weights to compensate for the occluded weed features, enabling the model to more effectively identify weed growth points under occlusion.

The CSMM structure uses different image patches to obtain multiscale features, and then DW convolution is used to learn the spatial dimensions and correlations of the channels. The outputs of the depthwise convolution are subsequently combined by pointwise (PW) convolution (1 ​× ​1). Ultimately, a two-layer fully connected network is employed to integrate the information from each channel, enhancing the connectivity between them. It compensates for loss in the occluded scene and emphasizes the weed regions in the image by utilizing the relationship between occluded and unoccluded weeds learned in the previous step.

With these improvements, the SRD-YOLO model demonstrates robust performance in identifying weed growth points under complex field conditions.

### 2.3 Experimental platform

The training and testing hardware platform of this study is the HP Z820 workstation, and the main hardware configuration is shown in Table S1. Uniform hyperparameters are used for different networks to avoid the influence of hyperparameters on the test results, as shown in Table S2.

### 2.4 Evaluation indicators

The performance of the model is analyzed using the keypoint similarity $L_{oks}$, the accuracy rate of each category (${Precision}_{kpt}$, $P_{kpt}$), the recall rate of each category (${Recall}_{kpt}$, $R_{kpt}$), the *F*1 score of each category, and the average precision value of each category ${AP}_{kpt}$ (${Average\;Precision}_{kpt}$), whereas the average value of all the categories ${AP}_{kpt}$ is ${mAP}_{kpt}$ [0.5]. These values are used as the model evaluation indices.

In keypoint detection, the object keypoint similarity $L_{oks}$ is used to validate the evaluation metrics for keypoint detection. The formula is as follows:

(2) $$L_{\text{oks}}=\frac{\sum_{i=1}^{i}\left[\exp^{\left(-\frac{d_{i}^{2}}{2s^{2}k_{i}^{2}}\right)}\delta \left(v_{i}>0\right)\right]}{\sum_{i=1}^{i}\delta \left(v_{i}>0\right)}$$

where i is the i-th element of among the labeled keypoints; $d_{i}$ is the Euclidean distance between the position of the detected keypoint and the position of the real keypoint; s is the scale factor of the detected target, whose value is the square root of the area of the detection frame of the detected target; $k_{i}$ is the attenuation constant of the keypoints; $v_{i}$ is the visibility of the i-th keypoint ($0\leq v_{i}\leq 2$), where $v_{i}$ is 0 for unlabeled, $v_{i}$ is 1 for labeled but invisible and $v_{i}$ is 2 for labeled and visible; and δ is a function calculated only for the labeled keypoints.

In addition, $P_{kpt}$, $R_{kpt}$, and *F*1 are calculated via Equations (3), (4), (5), respectively, and the *F*1 score is a comprehensive evaluation metric for keypoint detection.

(3) $$P_{kpt}=\frac{{TP}_{kpt}}{{TP}_{kpt}+{FP}_{kpt}}$$

(4) $$R_{kpt}=\frac{{TP}_{kpt}}{{TP}_{kpt}+{FN}_{kpt}}$$

(5) $$F1=\frac{2P_{kpt}R_{kpt}}{P_{kpt}+R_{kpt}}$$

where ${TP}_{kpt}$ is the predicted keypoint score above the $L_{oks}$ threshold (the $L_{oks}$ threshold is taken as 0.5) when the growth point is correctly recognized compared with the actual keypoint. The predicted keypoint above the $L_{oks}$ threshold when the non-growth point region is incorrectly recognized as a growth point is denoted as ${FP}_{kpt}$. ${FN}_{kpt}$ indicates an actual growing point situation that was missed because the corresponding predicted keypoint did not meet the required $L_{oks}$ threshold.

${AP}_{kpt}$ represents the area under the $P_{kpt}$-$R_{kpt}$ curve, while ${mAP}_{kpt}$ denotes the mean value of ${AP}_{kpt}$ across all categories, where *n* represents the number of categories. Both metrics are used to evaluate the overall performance of the model, with higher values indicating better performance. The calculation formulas are shown in Equations (6), (7).

(6) $$AP_{kpt}=\int_{0}^{1}P_{kpt}d\left(R_{kpt}\right)$$

(7) $$mAP_{kpt}=\frac{\sum_{i=1}^{n}AP_{i}}{n}$$

In addition, this study selects the FPS and the number of parameters as evaluation metrics to assess the model's image processing speed and size.

## 3 Results

### 3.1 Ablation and comparison experiments

To validate the effectiveness of the proposed improvements on the performance of the weed growth point detection algorithm, ablation experiments were conducted. The corresponding improvements were incrementally added to the YOLOv8-Pose network, and their effectiveness was analyzed by comparing the results.

The outcomes of the ablation experiments are presented in Table 2. Method A refers to the baseline model trained without data augmentation, whereas Method B incorporates augmentation techniques during training. As shown in Table 2, Method B achieves a 1.8 ​% improvement in ${mAP}_{kpt}$ compared with Method A, validating the effectiveness of data augmentation. In terms of structural optimization of the model, each introduced module contributed to enhancing model performance. Specifically, the integration of SEAM resulted in a 2.9 ​% increase in ${mAP}_{kpt}$, enabling the model to identify weed growth points more effectively under occluded conditions. The improvement of the RVB has reduced the model's parameters by 11.1M and increased its frames per second (FPS) by 8. This optimization not only achieves a more lightweight model but also significantly enhances the detection speed. The DWRM significantly improved the detection performance, achieving a 3.2 ​% increase in ${mAP}_{kpt}$ by effectively capturing multiscale contextual information. When all three modules were integrated into the network, ${mAP}_{kpt}$ reached 96.5 ​%, representing a 4.5 ​% improvement. Both the RVB and DWRM are lightweight modules, and the integration of these modules into the model results in a reduction in the number of parameters of the new model. The parameter count was reduced by 8.7M, and the FPS increased by 21 (frames/s), further enhancing the efficiency. These experimental results demonstrate the effectiveness of the four methods in improving model performance.

**Table 2:** Comparison of optimization methods. “Dat” denotes the use of data augmentation.

Methods	SEAM	RVB	DWRM	Dat	${mAP}_{kpt}$/%	*F*1/%	Parameters/M	FPS (frames/s)
A	×	×	×	×	90.2	85.6	43.5	148
B	×	×	×	✓	92.0	88.6	43.5	148
C	✓	×	×	✓	94.9	92.1	41.7	129
D	×	✓	×	✓	93.8	91.7	32.4	156
E	×	×	✓	✓	95.2	92.5	42.6	151
F	✓	✓	×	✓	95.3	92.7	31.0	153
G	✓	×	✓	✓	95.9	93.0	40.8	112
H	×	✓	✓	✓	95.5	92.9	36.7	155
I	✓	✓	✓	✓	96.5	94.6	34.8	169

To verify the statistical significance and stability of the proposed model improvements, we conducted six independent experiments comparing the SRD-YOLO model with the baseline YOLOv8-Pose model. Table S3 presents the results of various evaluation indicators of the six experiments. On the basis of these data, confidence interval estimation and paired sample t tests were performed. The statistical analysis results, summarized in Table S4, indicate that the SRD-YOLO model achieved significant improvements over the original model in all three key metrics (p ​< ​0.01). These findings demonstrate that the improvements in both accuracy and real-time performance achieved by SRD-YOLO are stable and reliable.

**Fig. 4:**
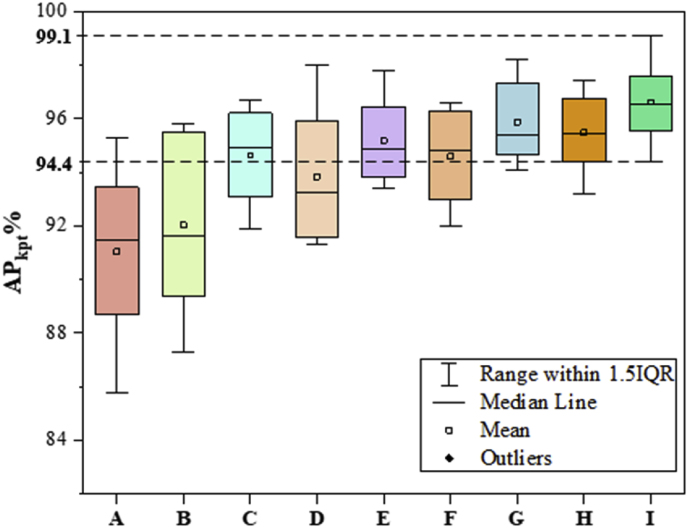
Ablation test chamber line diagram. The methods labeled A–I in the figure correspond to those listed in Table 2.

Fig. 4 presents a box-and-whisker plot comparison of the different models' ${mAP}_{kpt}$ values in the ablation experiments. Similarly, Method B outperforms Method A significantly in terms of both accuracy and robustness. However, both models exhibit relatively long box lengths and extended whiskers in the box plot, indicating significant variability in the ${AP}_{kpt}$ values for detecting different types of weeds. This variability suggests that Models A and B perform relatively inadequately when dealing with weeds that have small targets or indistinct growth points. Compared with Models A and B, the other models have improved detection performance, and the average ${AP}_{kpt}$ values for various types of weed detection are greater. Among them, Model I (SRD-YOLO) achieved the best results, with its maximum values (99.1 ​%), upper quartile, median, lower quartile, and minimum values (94.4 ​%) all outperforming the other models, indicating robust detection results for various weed types.

A visual comparison of the detection results between Methods B and C is shown in Fig. S2 (A) and (B). Method C, which incorporates SEAM, exhibited superior detection performance under occluded conditions, successfully identifying the growth points of Ei. obscured by corn leaves. Fig. S2 (C) and (D) show the detection effects of Methods B and E. Method E, which integrates the DWRM, demonstrated excellent detection performance for densely clustered weeds, successfully identifying densely growing weeds such as Ep. and Ea. in (D).

### 3.2 Comparison with the other models

To evaluate the performance of the proposed algorithm, comparative experiments were conducted against five keypoint detection algorithms: YOLOv7-Pose, YOLOv5-Pose, YOLOv6-Pose, YOLOv8-Pose and SRD-YOLO, as shown in Table 3. The primary focus of this study is the recognition of weed growth points. The evaluation metrics include the *F*1 score, ${mAP}_{kpt}$, and training curves to assess the detection capability of the model, as well as FPS to evaluate its real-time performance.

**Table 3:** Comparison of test results for different detection networks.

Module	Corn	Ei.	Ep.	Ea.	Gb.	Pl.	Sg.	Ow.	${mAP}_{kpt}$/%	FPS (frames/s)
	*F*1/%	*F*1/%	*F*1/%	*F*1/%	*F*1/%	*F*1/%	*F*1/%	*F*1/%		
YOLOv5-Pose	93.9	89.1	85.9	88.2	88.4	89.7	88.4	91.3	89.7	127
YOLOv6-Pose	90.1	87.8	87.7	82.1	84.4	83.0	92.2	84.4	91.2	137
YOLOv7-Pose	93.1	87.1	85.3	88.5	89.1	89.4	88.3	84.9	88.3	135
YOLOv8-Pose	91.4	92.1	86.5	80.7	85.3	87.1	94.9	85.4	92.0	148
SRD-YOLO	95.6	96.5	93.6	91.7	93.4	93.5	97.2	92.3	96.5	169

**Fig. 5:**
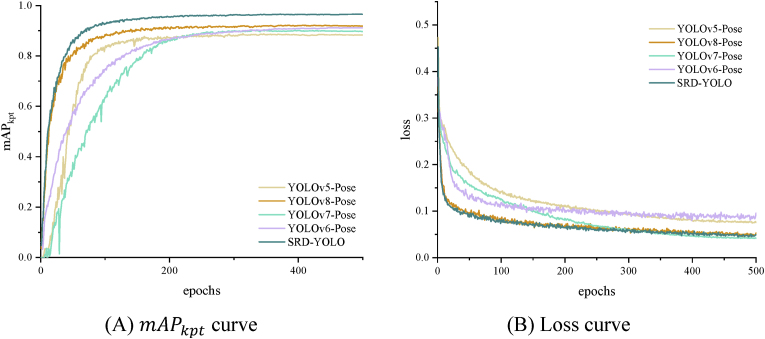
Training curves.

The keypoint loss function used in this study calculates the Euclidean distance between the predicted keypoints and the true keypoints using the mean square error (MSE) loss method. This approach quantifies the difference between the two, thereby assisting the network in accurately locating and identifying keypoints in the image. The MSE calculation formula is shown in Formula (8).

(8) $$MSE=\frac{1}{n}\sum_{i=1}^{n}{\left(y_{ri}-y_{i}\right)}^{2}$$

where n represents the number of samples, $y_{ri}$ denotes the i-th element in the predicted keypoints, and $y_{i}$ denotes the i-th element in the ground truth keypoints. The closer the predicted keypoints are to the ground truth, the smaller the mean squared error. This is reflected in the loss curve, where a lower loss value indicates a closer match between the predictions and ground truth. Fig. 5 shows the training curves.

As shown in Table 3 and Fig. 5(A), YOLOv8-Pose outperforms YOLOv5-Pose, YOLOv6-Pose, and YOLOv7-Pose in terms of detection performance, with ${mAP}_{kpt}$ values improved by 2.3 ​%, 0.8 ​%, and 3.7 ​%, respectively, and FPS increased by 21, 11, and 13, respectively. Therefore, we chose to improve upon YOLOv8-Pose. For densely distributed weeds with difficult-to-locate growth points, such as Ep., Ea., Gb., and Pl., the *F*1 scores for detection using the first four networks listed in Table 3 are all less than 90 ​%. In contrast, the SRD-YOLO model achieves *F*1 scores exceeding 90 ​% for keypoint detection across corn and all seven weed species. Overall, the improved SRD-YOLO achieves the highest detection accuracy, with ${mAP}_{kpt}$ values exceeding those of YOLOv5-Pose, YOLOv6-Pose, YOLOv7-Pose, and YOLOv8-Pose by 6.8 ​%, 5.3 ​%, 8.2 ​%, and 3.5 ​%, respectively.

From the loss curves in Fig. 5(B), it is evident that YOLOv8-Pose and SRD-YOLO rapidly decrease within the first 100 epochs and gradually stabilize, whereas YOLOv5-Pose and YOLOv7-Pose exhibit slower declines, stabilizing after approximately 250 epochs. Although YOLOv6-Pose stabilizes within 100 epochs, it has the highest loss value. Among these, SRD-YOLO converges the fastest, with a slightly higher loss value than YOLOv7-Pose but a lower value than the other three networks achieve, indicating more accurate localization of growth points. In terms of detection speed, SRD-YOLO achieves an FPS of 169 (frames/s), which is 42, 32, 34, and 21 (frames/s) higher than those of the other four networks. Taken together, the SRD-YOLO model developed in this study demonstrates superior overall performance.

### 3.3 Detection of growth points in the presence of occlusion

**Fig. 6:**
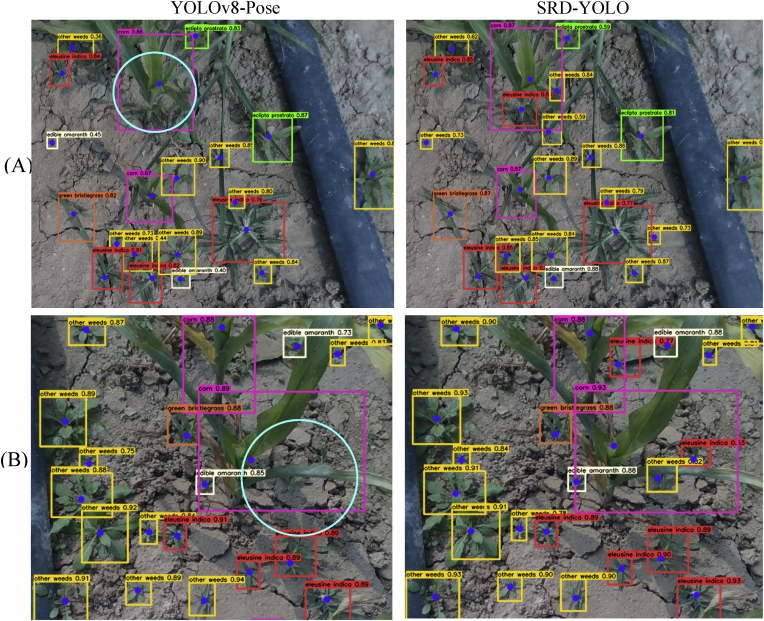
Visual comparisons of YOLOv8-Pose and SRD-YOLO for occlusion.

We evaluated the performance of SRD-YOLO under occlusion conditions, and two visual comparisons of the detection results for YOLOv8-Pose and SRD-YOLO are shown in Fig. 6. Combined with the data in Table 2, the ${mAP}_{kpt}$ of the model after the introduction of SEAM is improved by 2.9 ​% compared with that of YOLOv8-Pose. As illustrated in Fig. 6, YOLOv8-Pose exhibited missed detections of weeds and their growth points in scenarios where corn and weeds were mutually occluded, failing to identify weeds obscured by corn. In contrast, SRD-YOLO successfully identified Ei. and Ow. occluded by corn and accurately localized their growth points in Fig. 6(A)–as well as in Fig. 6(B). Moreover, the average confidence level of SRD-YOLO is above 0.85. Therefore, SRD-YOLO has a better detection effect in the case of occlusion

### 3.4 Growth point detection in dense clusters of weeds

**Fig. 7:**
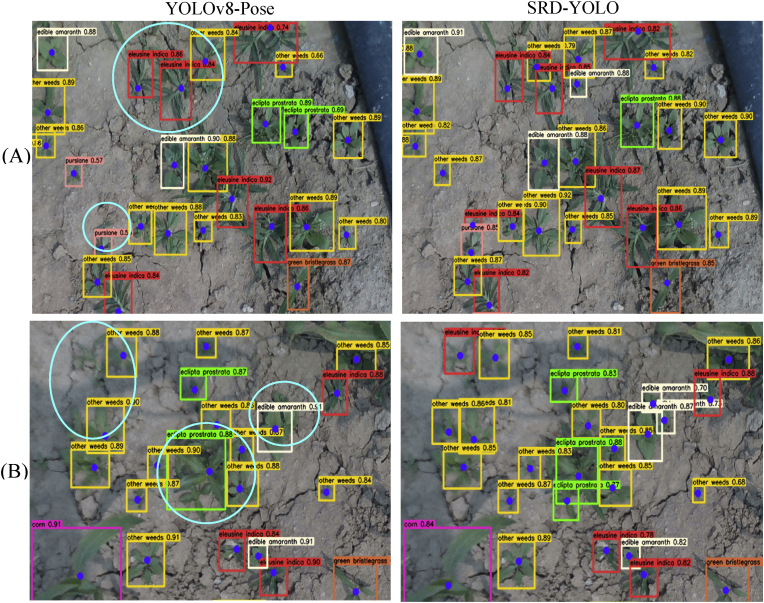
Visual comparisons of YOLOv8-Pose and SRD-YOLO.

The models were also tested under conditions of diverse weed species and uneven density distributions, with Fig. 7 presenting visual comparisons of the detection results between YOLOv8-Pose and SRD-YOLO. Under dense weed conditions, YOLOv8-Pose resulted in missed detections, particularly for some smaller, densely distributed weed targets such as Ea. and Ow. Table 3 shows the *F*1 scores of YOLOv8-Pose for Ea. and Ow. were 80.7 ​% and 85.4 ​%, respectively, which were lower than those of the other weed categories. SRD-YOLO had *F*1 scores above 90 ​% for all weed types. Fig. 7 shows that SRD-YOLO successfully recognizes the classification of weeds and the localization of growth points in a dense weed situation with an average confidence level above 0.83. The densely growing Ei. and Ow. in (A) were successfully identified and accurately localized to their growth points, as were Ep., Ea., and Ow. in (B), which were also effectively recognized and precisely mapped to their respective growth points. Therefore, SRD-YOLO can achieve a better detection effect in the case of dense clusters of weeds.

### 3.5 Detection of growth points under different light intensities

The operation time of the precision weeding robot spans 24 ​h a day. To ensure detection effectiveness across all time periods, this study constructed a dataset comprising corn field images captured under different lighting conditions: strong daylight, weak daylight, and nighttime illumination (LED light source).

**Fig. 8:**
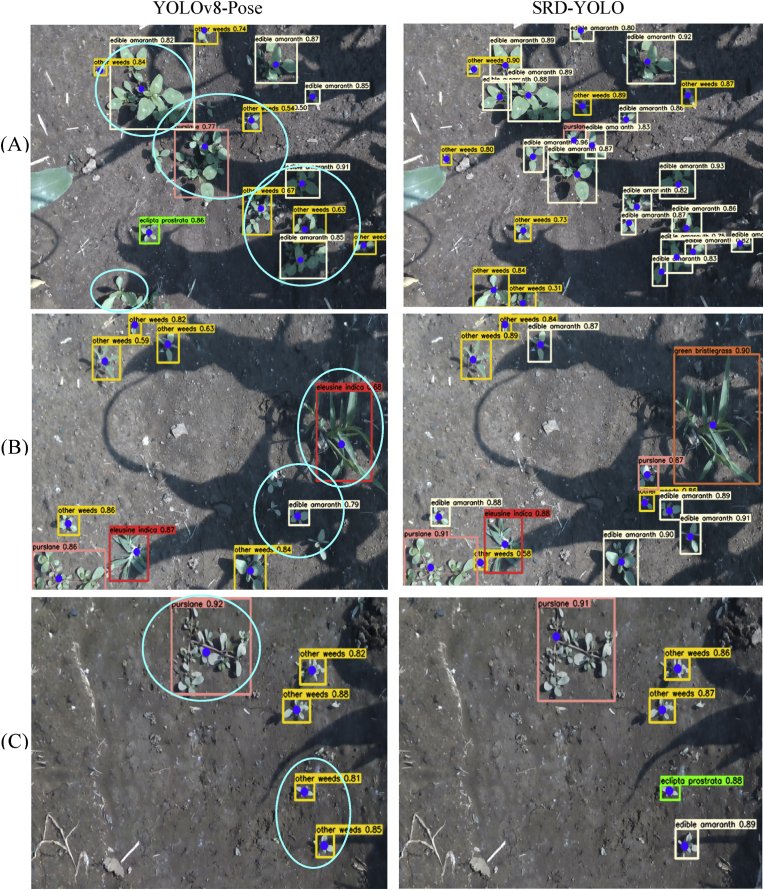

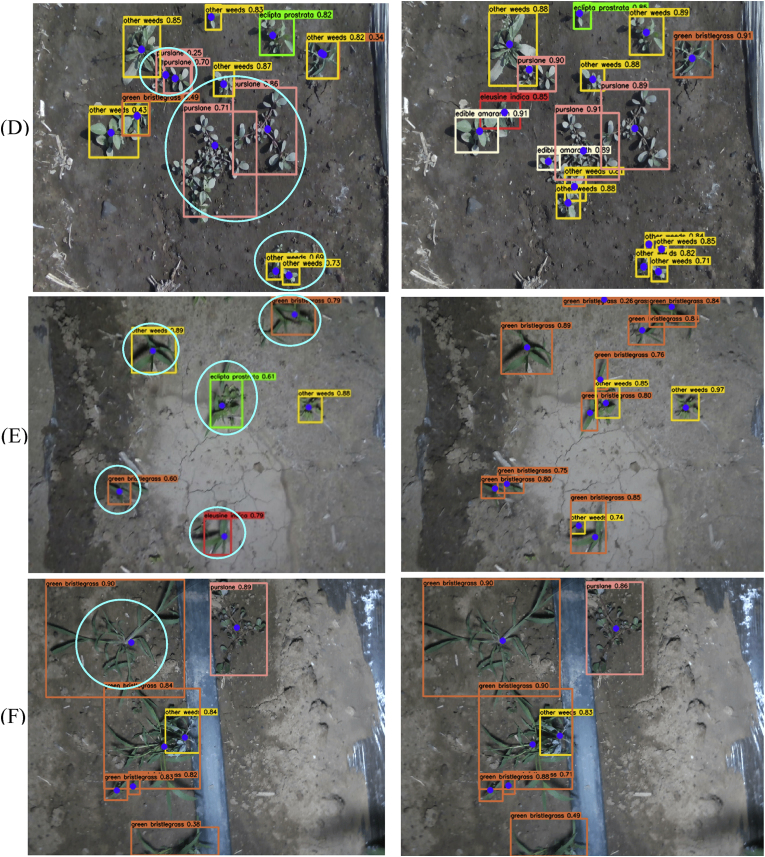
Detection visualization under different lighting conditions. (A)–(B) show strong daylight intensity conditions. (C)–(D) show weaker daylight intensity conditions. (E)–(F) show nighttime light intensity conditions.

Under strong daylight conditions, the images contained numerous shadows, which weakened the features of weeds in shadowed areas. As shown in Fig. 8(A) and (B), compared with SRD-YOLO, YOLOv8-Pose missed 16 weeds, including 8 Ea., 1 Pl., and 4 Ow. in Fig. 8(A)–as well as 2 Ea., 1 ​Pl. and 2 Ow. in Fig. 8(B). Additionally, in Fig. 8(B), YOLOv8-Pose misidentifies Gb. as Ei., and growth point localization deviations occur. In contrast, SRD-YOLO correctly classifies Gb. and accurately locates its growth points.

When the daylight was weaker, shadows were largely absent, and lighting had a minimal impact on the detection performance, as depicted in Fig. 8(C) and (D). However, when weed growth is dense, YOLOv8-Pose results in both missed and false-positive results. Redundant frames with confidence scores below 0.5 are also shown in Fig. 8(D). Furthermore, owing to the high number of stems and leaves of Pl., YOLOv8-Pose shows a deviation in the localization of growth points in Fig. 8(C) and (D). Although SRD-YOLO exhibits slight localization bias for the growth points of Pl. in Fig. 8(D), it significantly reduces the miss detection and false detection rates under dense conditions.

Night images were captured under LED light sources, where uneven illumination rendered the features of the weeds and their growth points less distinguishable, as shown in Fig. 8(E). YOLOv8-Pose exhibited a higher miss rate when detecting smaller targets. Moreover, when the shape of the weeds was irregular, the growth point localization became inaccurate. This phenomenon was evident for Gb. and Pl. in Fig. 8(F), where deviations in growth point localization were observed. SRD-YOLO also exhibits slight deviations in detecting Pl. in Fig. 8 (F), but its overall detection performance is significantly superior to that of YOLOv8-Pose.

In summary, SRD-YOLO demonstrated superior detection performance across varying lighting intensities. The confidence scores of most weed detection boxes exceeded 0.8, while the miss rates and false-positive rates were significantly reduced. Furthermore, SRD-YOLO exhibited no notable deviations in growth point localization under any lighting condition. This establishes a solid foundation for achieving precise weed suppression during the 24-h continuous operation of precision weeding robots.

### 3.6 Field test

**Fig. 9:**
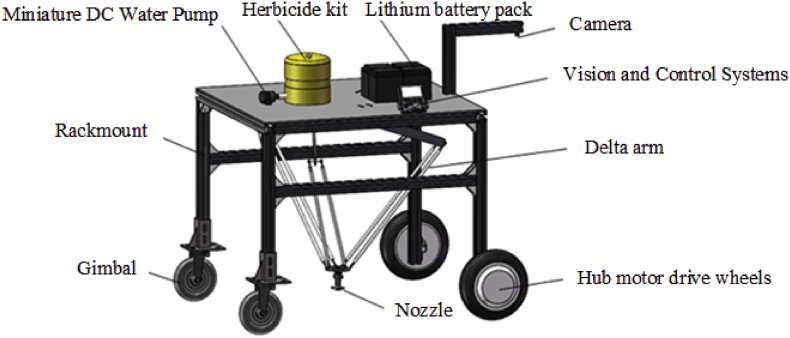
Schematic structure of the parallel arm precision weeding robot.

To validate the feasibility of the proposed method, the SRD-YOLO model was deployed on a delta-arm precision weeding robot for experimental verification. The structure of the precision weeding robot is shown in Fig. 9. The hardware of the vision system consists primarily of the Raspberry Pi camera V2 and the Jetson Orin NX edge computing platform. The camera is equipped with a SONY IMX219 image sensor, with a maximum resolution of 3280 ​× ​2646. The Jetson Orin NX features an NVIDIA Ampere architecture GPU, an Arm Cortex-A78AE CPU, and 16 ​GB of memory.

The robot uses a camera to capture images of the cornfield, which are then sent to the vision system for processing. The SRD-YOLO model is employed to classify corn and weeds while precisely identifying and locating the growth points. These coordinates are subsequently transmitted to the control system and used to guide the delta arm to spray herbicide precisely on the targeted points.

**Fig. 10:**
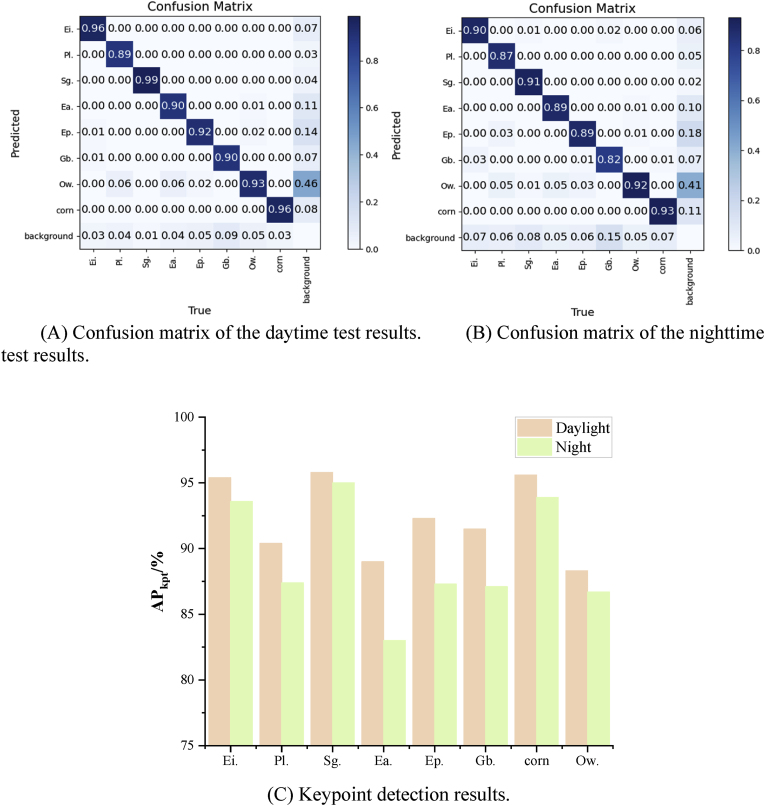
Results of the field test.

Field experiments were conducted in the corn test field at the Panhe Campus of Shandong Agricultural University. The corn varieties included three widely cultivated types, namely, Denghai 605, Zhengdan 958, and Nongda 108, each planted in separate experimental plots. The crop row spacing was 60 ​cm, the plant spacing was 16 ​cm, and the corn growth stage was between the 3rd and 5th leaf stages. The experiments were carried out both during the day and at night, and a field test of the precision weeding robot is shown in Fig. S3. During the field test, the precision weeding robot operated at a walking speed of 0.3 ​m/s, capturing a total of 350 field images—192 during the day and 158 ​at night. The images included 3325 plant samples, with 1815 in the daytime images and 1510 in the nighttime images. The distribution of sample counts is shown in Table 4. To provide a more detailed demonstration of the proposed algorithm's detection performance for corn and various weed types, a confusion matrix and bar charts of the keypoint detection results were generated, as shown in Fig. 10.

**Table 4:** Distribution of the number of samples in field images.

Classification	Number of vegetative samples	
	Daylight	Night
Ei.	206	186
Pl.	128	101
Sg.	124	107
Ea.	129	114
Ep.	325	287
Gb.	168	145
Ow.	526	421
Corn	209	149

In Fig. 10(A) and (B) present the normalized confusion matrix for weed detection and classification. The classification performance for Pl. and Gb. was relatively poor. During the daytime testing, 6 ​% of the other weed species were misclassified as Pl., and 9 ​% of the background areas were incorrectly identified as Gb. During the nighttime testing, 5 ​% of the other weed species were misclassified as Pl., and 15 ​% of the background regions were misclassified as Gb. These misclassifications may be attributed to the visual similarity between certain weed species and Pl., as well as the morphological resemblance between Gb. and some background textures, such as corn crop residues, as shown in Fig. S4. These similar features may confuse the feature extraction module and lead to incorrect predictions by the model. The daytime test results outperformed the nighttime test results, particularly for Ei., Sg., and Gb., which showed improvements of 6 ​%, 8 ​%, and 8 ​%, respectively. This discrepancy may be attributed to the greater likelihood of pixel blurring caused by camera shake in nighttime environments, which increases the difficulty of detecting irregularly shaped weeds. The percentage of Ow. in the false detection samples of both confusion matrices was the highest for other weeds because they contained multiple weed types with different shapes, which made detection difficult.

In terms of keypoint detection, Fig. 10(C) shows the results of growth point detection for various weed types during the daytime and nighttime. Keypoint detection results of Pl., Ea. and Ow. are relatively poor. This may be attributed to the dense and compact cotyledons of Pl., with the growth point located at the root, which often leads to occlusion issues and increases the difficulty of detection. The Ow. comprises multiple weed species, most of which are small in size and lack prominent growth points. For Ea. species, the growth point is located at the apical stem. However, some individuals are small, and their growth points are wrapped by cotyledons, making the features less distinguishable, as shown in Fig. S5. These factors collectively make accurate detection of growth points more challenging.

Fig. S6 shows example detection results during the operation of the experimental platform. The proposed model achieves a processing speed of 24 frames per second (FPS) on the Jetson Orin NX edge computing platform, with an average inference time of 41.7 ​ms per image. Although deployed on an edge computing platform with limited computational resources, resulting in a decrease in image processing speed, the model still achieves real-time detection performance at a traveling speed of 0.3 ​m/s. The experimental results demonstrate that the proposed algorithm, when deployed on mobile devices, achieves satisfactory performance in weed classification and growth point localization. This provides a feasible method for identifying weed growth points in resource-constrained precision weeding systems for field applications.

## 4 Discussion

Most research in the field of weed detection focuses on the overall detection and segmentation of weeds [6,[8], [9], [10], [11]], which only allows for the identification of whole or partial weeds, making it difficult to locate growth points precisely. Wang et al. [30] proposed a lightweight model based on an improved YOLOv3 [31] for identifying the growth points of the main stems of cotton. Their approach determined the growth point's range by locating portions of the main stem through bounding box detection, but it fails to accurately identify the precise location of the growth points. Arsa et al. [32] developed an encoder–decoder-based convolutional neural network that combines heatmaps and segmentation branches to perform weed growth point detection. Their method achieved a detection rate of 85.05 ​% and a precision of 86.41 ​% on the weed growth point detection task. In contrast, our keypoint-based growth point detection method achieves an F1 score of over 90 ​% for the growth points of various weed species, demonstrating superior accuracy.

Despite its advantages, the proposed method in this study has certain limitations. Motion blur issues are more likely to occur during nighttime operation. In future work, we will explore image restoration techniques to address the problem of nighttime motion blur.

We propose a keypoint-based growth point detection method for corn fields. The confusion matrix obtained during the test indicates that the classification performance for Pl. and Gb. was suboptimal. This is likely due to visual similarities between these weeds and certain Ow. or background textures, which may cause confusion in the feature extraction module. In future work, we aim to incorporate more robust background modeling and attention mechanisms to mitigate such misclassifications. The method is capable of detecting growth points for corn and multiple types of weeds, providing technical support for the weed recognition system of precision weeding robots. The main conclusions of this study are as follows:

A dataset suitable for North China was constructed, comprising thousands of images of weeds in corn fields under various scenarios, including corn–weed occlusion, uneven weed distribution, varying light intensities, and nighttime conditions. To enable growth point detection in these complex scenarios, the SEAM, RepViT block, and DWRM are integrated into YOLOv8-Pose, enhancing the model's feature recognition ability and detection speed. The experimental results in Table 2 show that the proposed method achieves an ${mAP}_{kpt}$ of 96.5 ​%, with *F*1 scores for corn and various weeds exceeding 90 ​%. The FPS reached 169 (frames/s). Compared with the baseline methods, ${mAP}_{kpt}$ improved by 4.5 ​%, FPS increased by 21 (frames/s), and the model's parameter size was reduced by 8.7M. These improvements demonstrate that the method achieves high accuracy while consuming less memory.

The experimental results demonstrate that the proposed method meets practical requirements for weed control in corn fields during the 2nd–5th leaf stage. It is capable of detecting the growth points of companion weeds, laying a technical foundation for precision weed control robots. It is hoped that this study will contribute to future research efforts and promote sustainable development in agriculture.

## Author contributions

M.L.: Conceptualization, methodology, writing—original draft, visualization, and funding acquisition. X.X.: writing—review and editing, charting. T.T. and M.S.: Investigation and data curation. Z.S. and F.T.: Validation and resources. Y.Y.: Supervision and funding acquisition.

## Funding

This work was supported in part by the Tibet Shigatse Science and Technology Projects (No. RKZ2024ZY-03), the Shandong Province Modern Agricultural Industry Technology System, China (No. SDAIT-18-06), the China Agriculture Research System of MOF and MARA (No. CARS-18-ZJ0402), and the 10.13039/100014717National Natural Science Foundation of China (No. 32001419).

## Data availability

A portion of the dataset used in this study, along with the trained weights of the SRD-YOLO model, is available at https://github.com/guhnb/SRD-YOLO.git. The complete dataset is available upon reasonable request by contacting the corresponding author.

## Declaration of competing interest

The authors declare that they have no known competing financial interests or personal relationships that could have appeared to influence the work reported in this paper.
