# Supplementary material for: A keypoint-based method for detecting weed growth points in corn field environments
Source: Plant Phenomics. 2025 Jun 20;7(3):100072. doi: 10.1016/j.plaphe.2025.100072 (PMC12709958; doi:10.1016/j.plaphe.2025.100072)
Supplement: Multimedia component 1 [file mmc1.docx]

**Supplementary Materials**

**Fig.S1.** **Images in different conditions.**

(B) Method C

(A) Method B

(C) Method B

(D) Method E


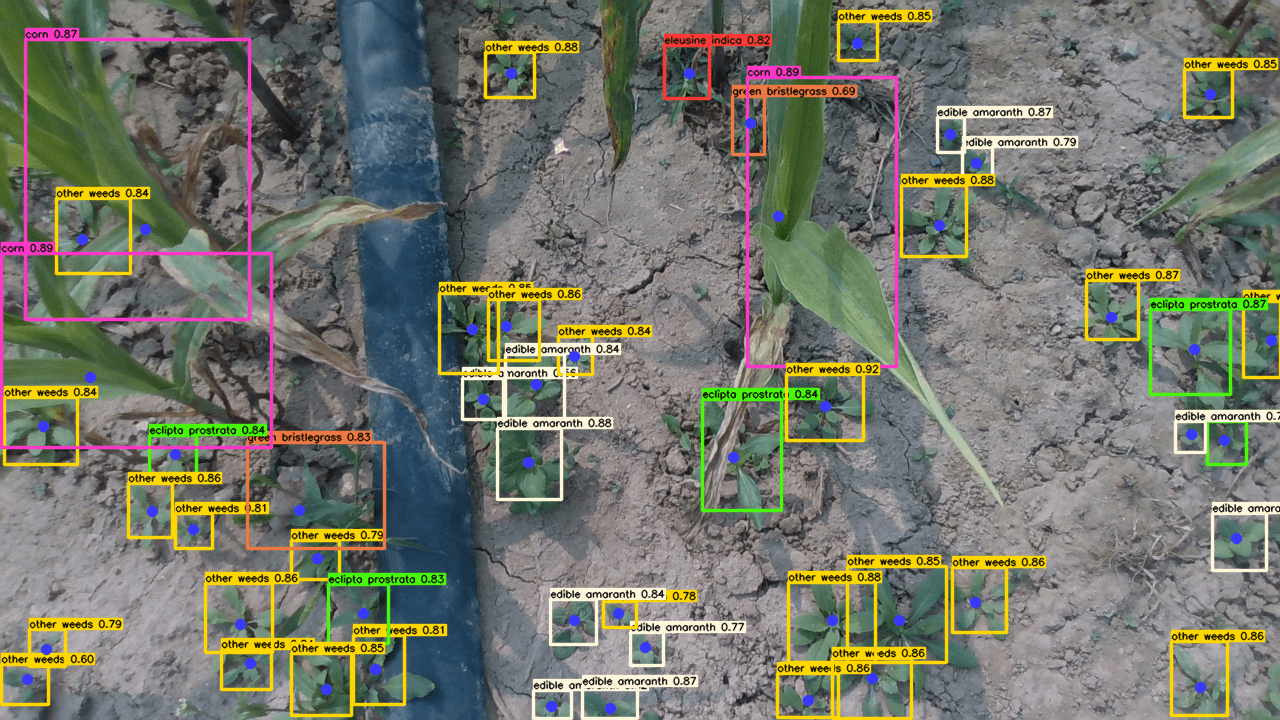

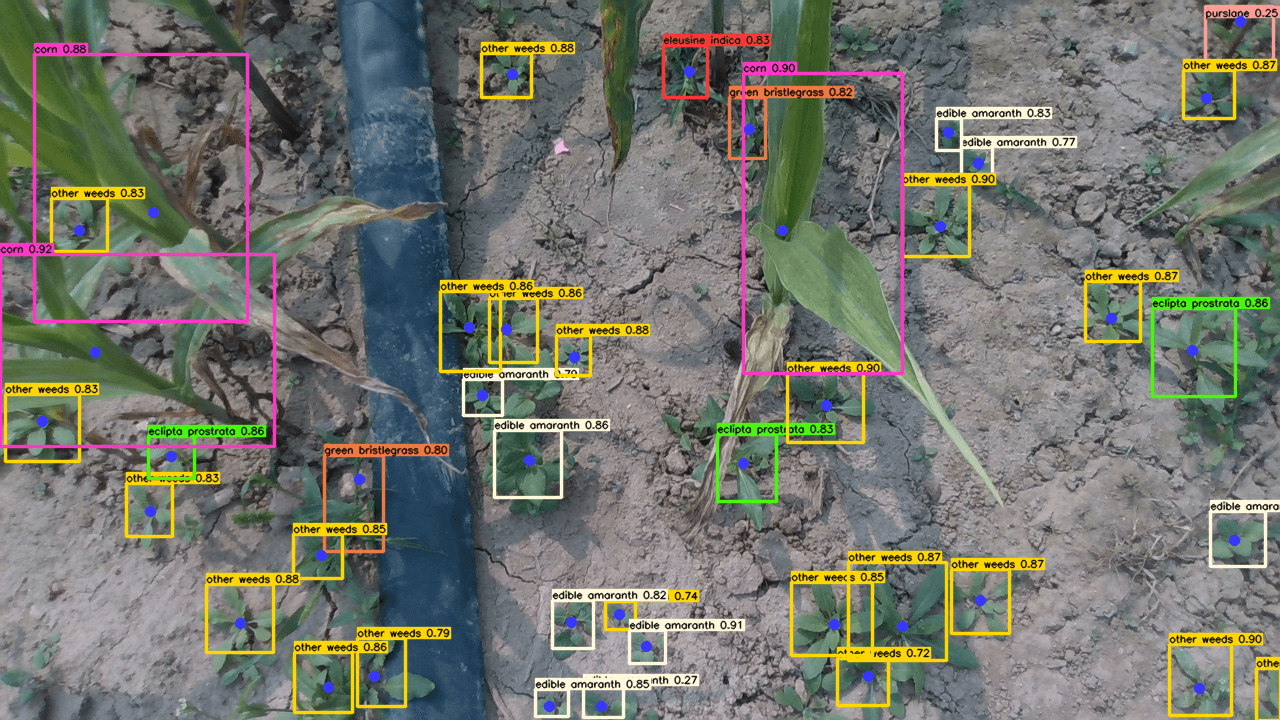

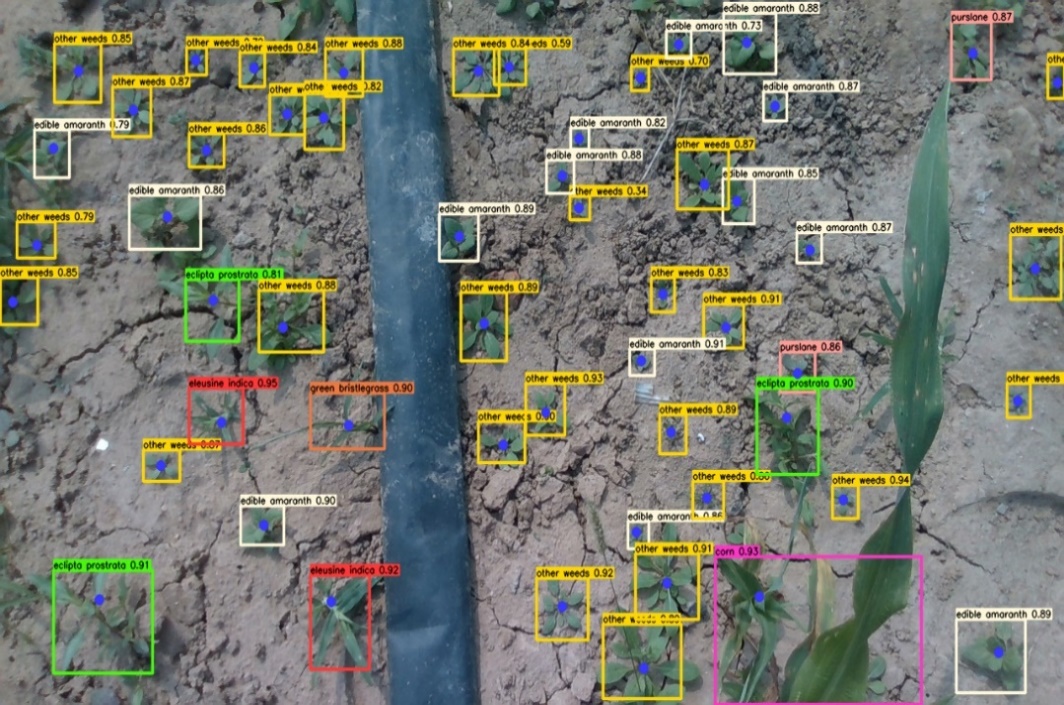

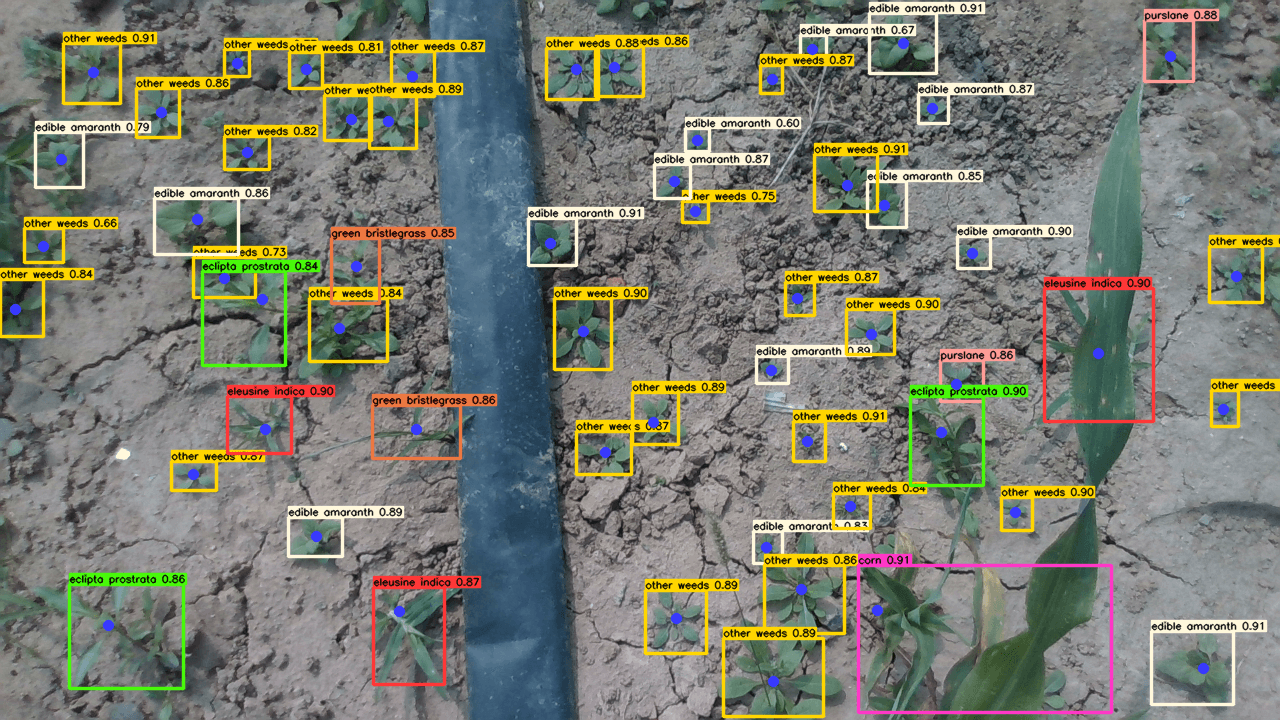


(D) Weak daylight

(E) Night light

(A) Obscured


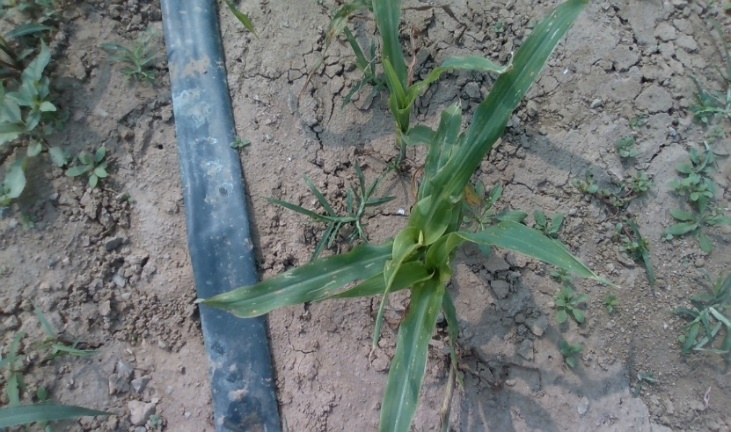

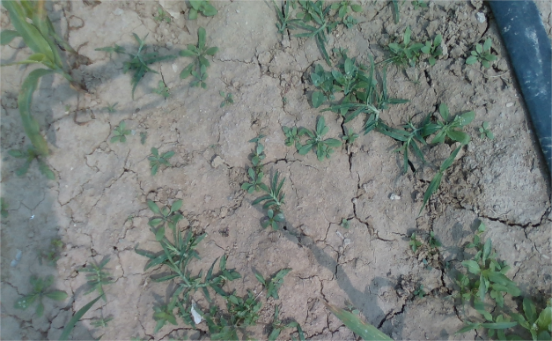

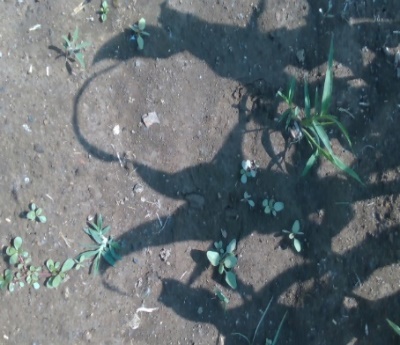

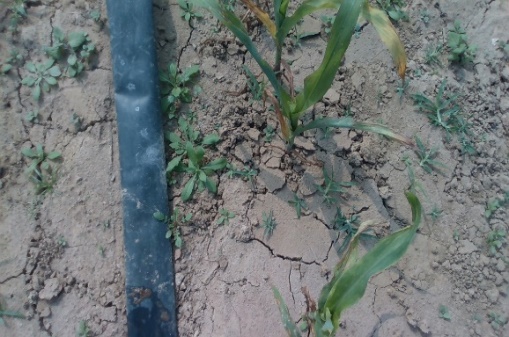


(B) Uneven distribution

(C) Strong Daylight


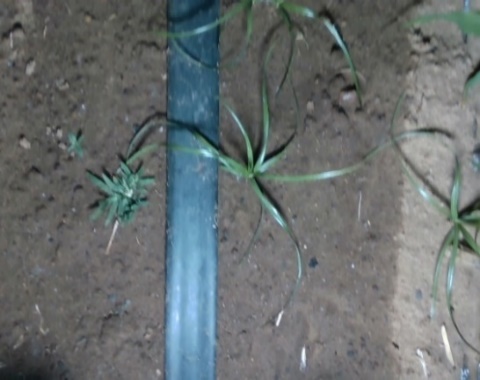

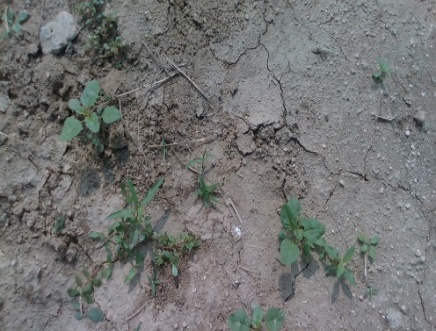


(F) Field obstructions

**Fig.S2.** **The visualization of the detection effects of method B, method C and method E.** The circled regions highlight missed detections caused by two primary factors: occlusion from overlapping corn leaves (figure (A) and (B)) and dense clustering of weeds (figure (C) and (D)), which complicates keypoint localization and instance discrimination.


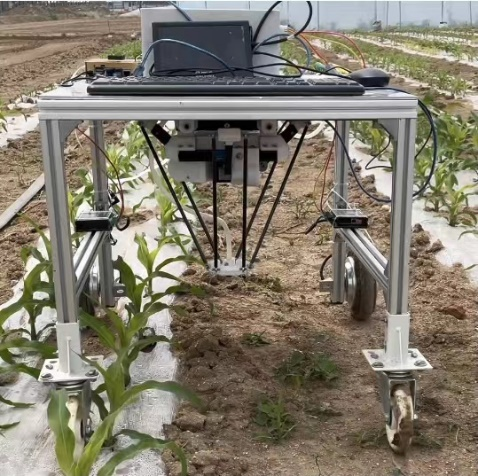

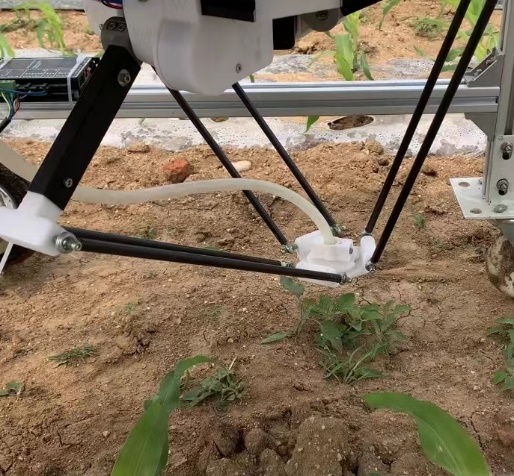


(A) Photograph of the robot walking in the field (B) End actuator nozzle reaches the weed position

**Fig.S3.** **Images of field test.**


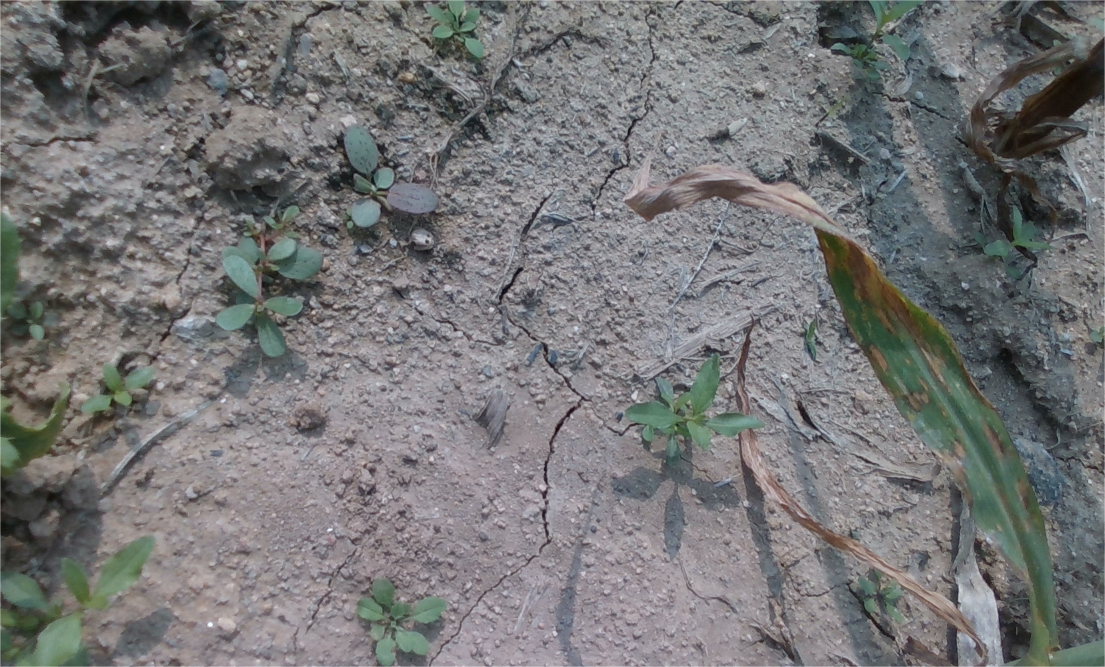

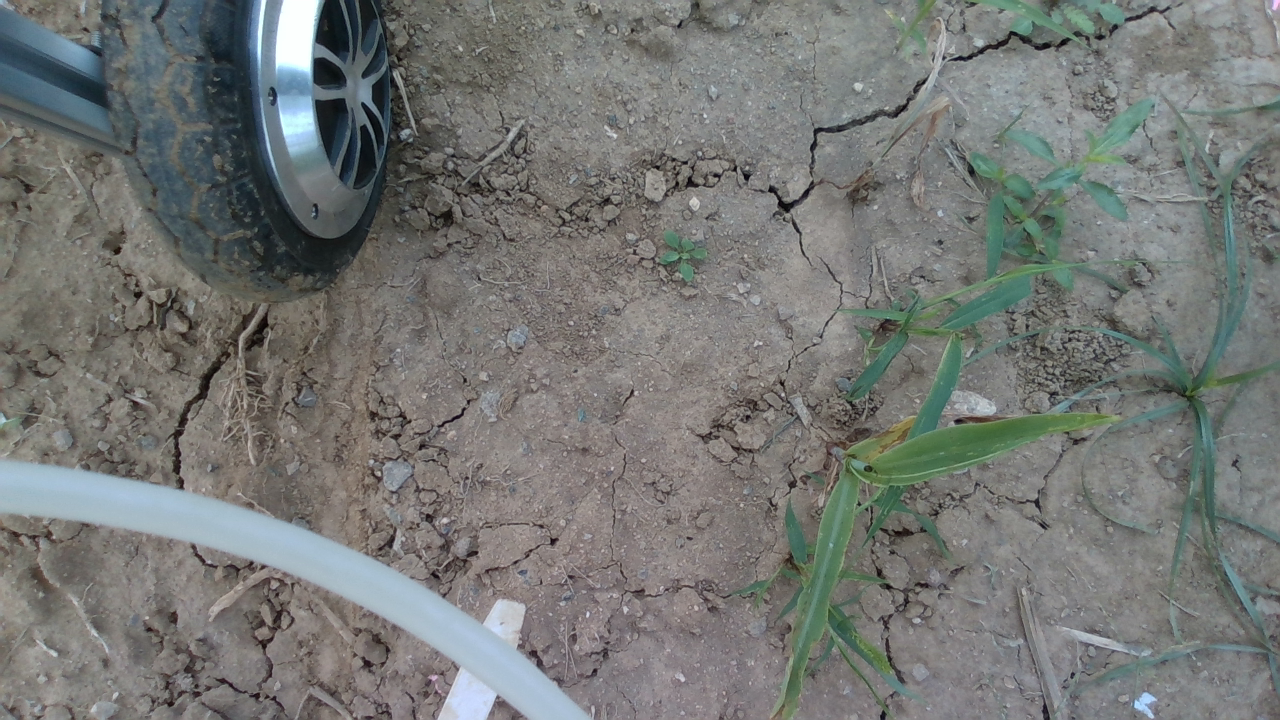


(A) Pl. and Ow. (B) Gb. and corn seedlings

**Fig.S4.** **Field image of weeds.** The yellow box is Pl., the red box is Ow., the blue box is Gb., and the purple box is corn seedlings.


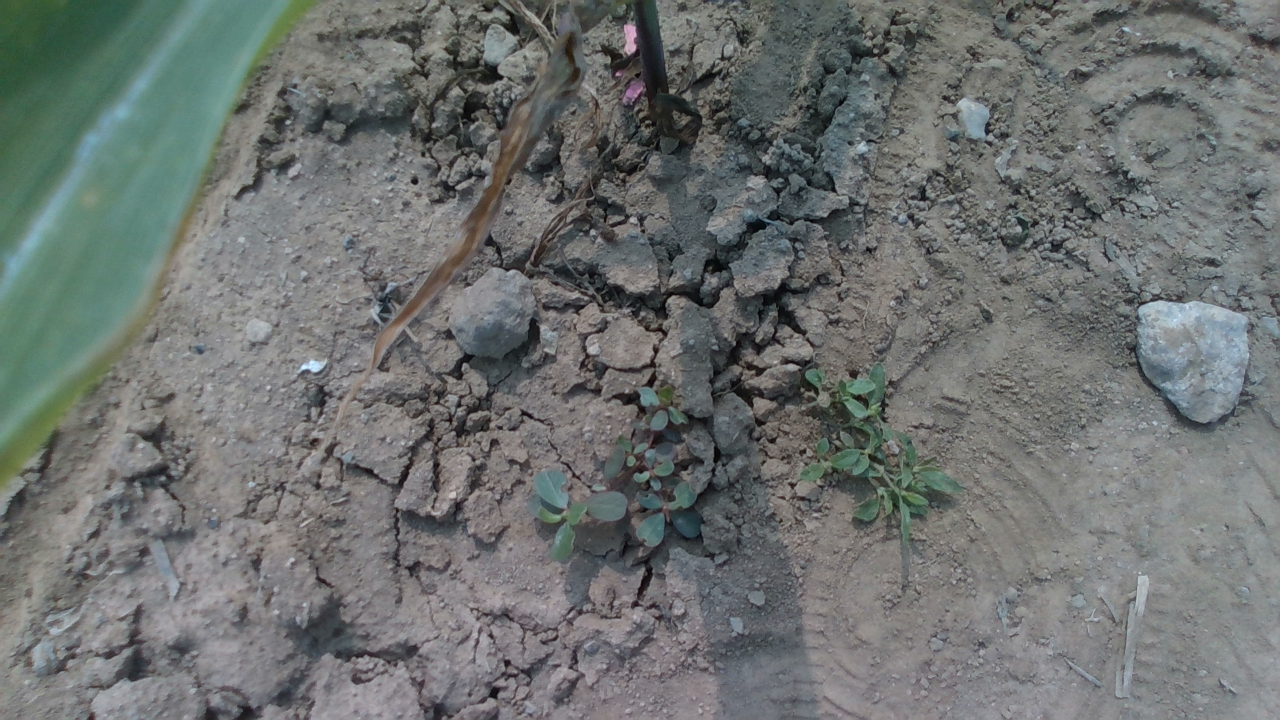

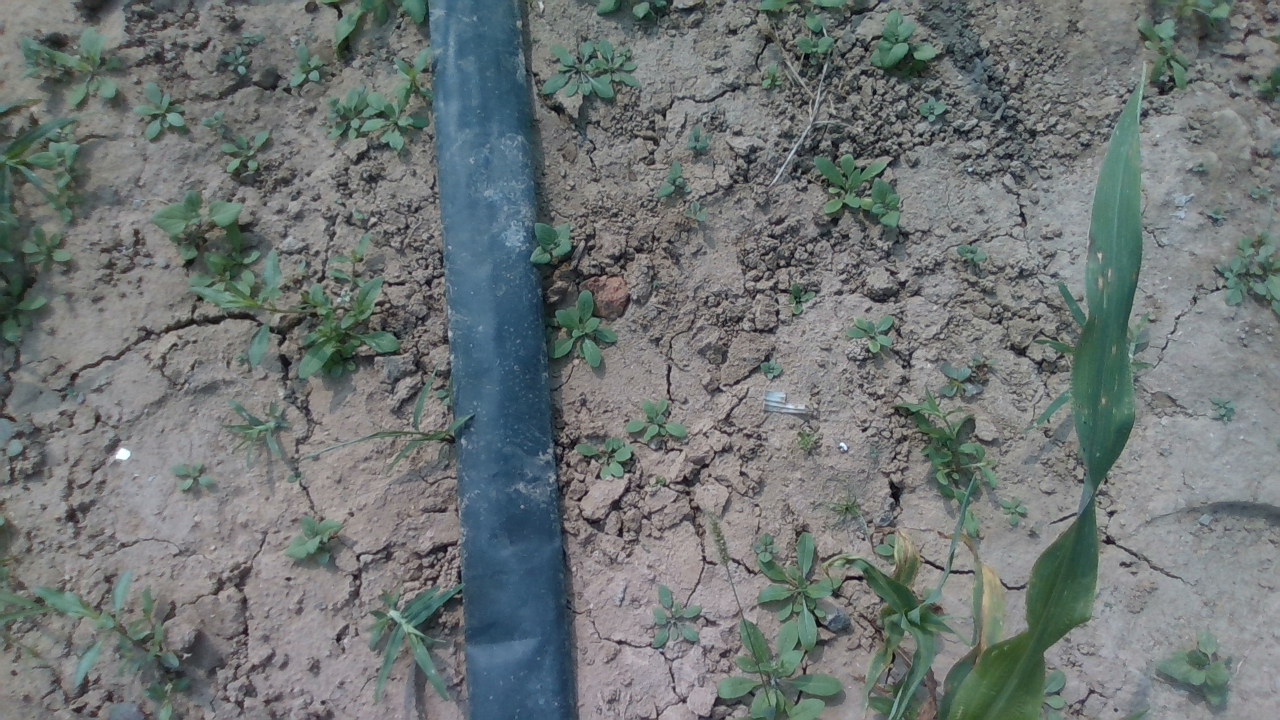

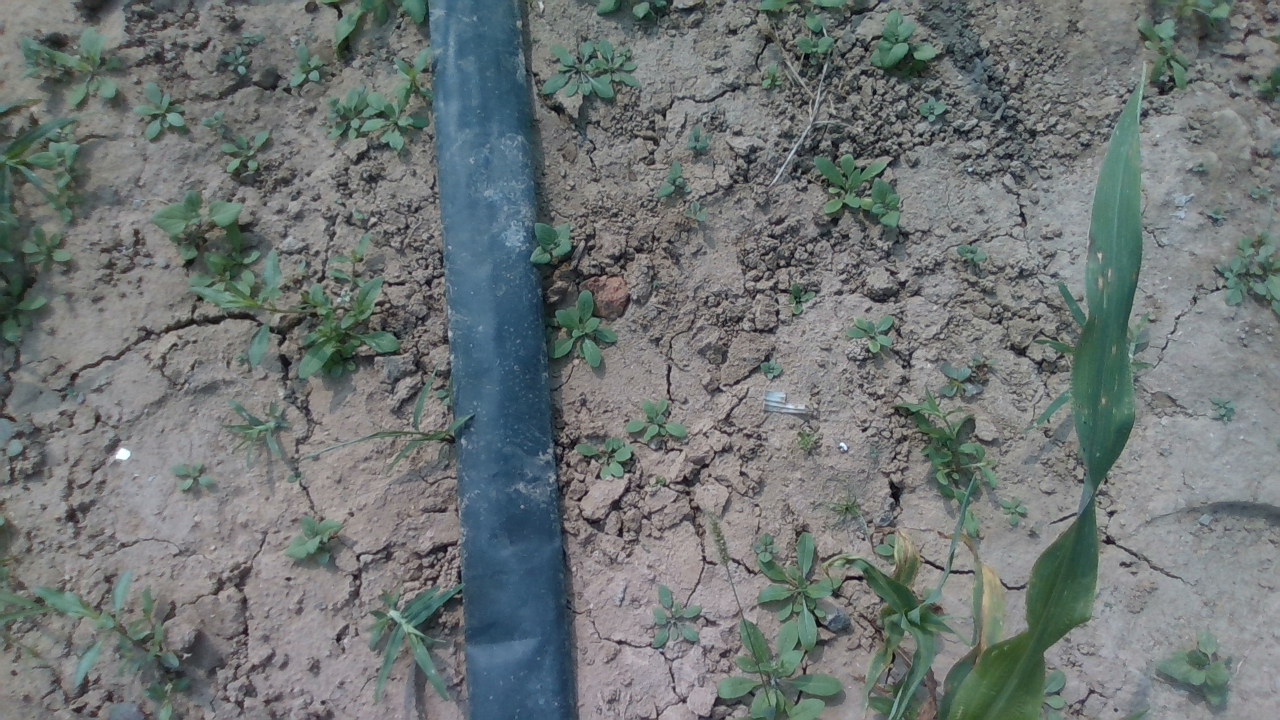


(A)Pl. (B)Ea. (C)Ow.

**Fig.S5.** **Example of a weed image.** The yellow circles indicate growth point positions.

**Fig.S6.** **Example Images of Detection Results from Field Experiments.**

Daylight


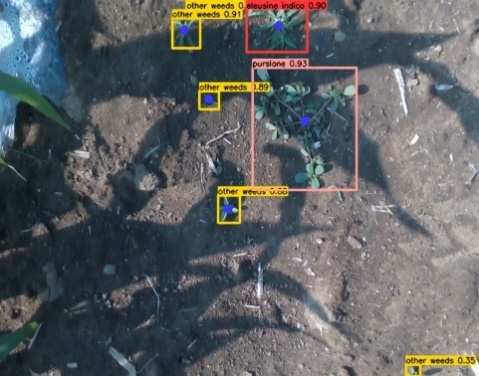

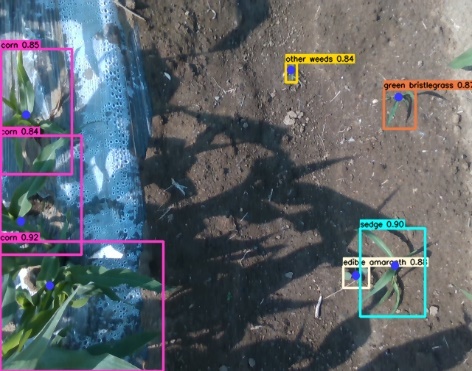

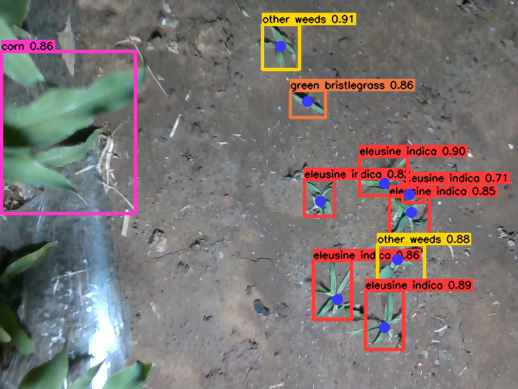

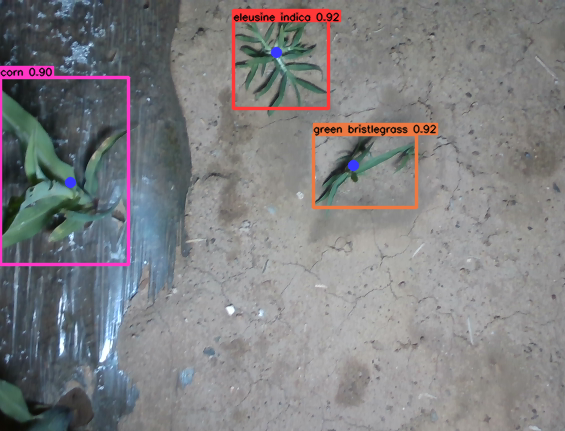


Nightlight

**Table S1.** Hardware and software parameters.

| Configuration | Parameter |
| --- | --- |
| CPU | Intel Xeon E5-2620 |
| RAM | 16 G |
| GPU | GeForce RTX 2080 Ti |
| Display memory | 11 G |
| Accelerated environment | CUDA 11.0 and CUDNN 7.1 |
| Operating system | Windows 10.0 |
| Development environment | Python 3.9.10 and PyTorch 1.2.0 |

**Table S2.** Experimental settings.

| Parameter | Value |
| --- | --- |
| Size of input images | 640×640 |
| Batch | 16 |
| Epoch | 500 |
| Initial learning rate | 0.01 |
| Optimizer | SGD |
| Momentum | 0.937 |

**Table S3.** **Independent training results.**

| Number of trials | YOLOv8-Pose | | | SRD-YOLO | | |
| --- | --- | --- | --- | --- | --- | --- |
|  | ${m\mathrm{AP}}_{kpt}$/% | F1/% | FPS (frames/s) | ${m\mathrm{AP}}_{kpt}$/% | F1/% | FPS (frames/s) |
| Run 1 | 92.0 | 88.6 | 148 | 96.5 | 94.6 | 169 |
| Run 2 | 91.8 | 89.1 | 143 | 96.6 | 94.7 | 165 |
| Run 3 | 92.4 | 90.3 | 145 | 96.0 | 94.1 | 170 |
| Run 4 | 91.6 | 88.5 | 149 | 96.3 | 94.2 | 168 |
| Run 5 | 92.3 | 90.1 | 142 | 96.2 | 94.1 | 166 |
| Run 6 | 92.5 | 90.3 | 146 | 96.6 | 94.7 | 163 |

**Table S4.** **Statistical analysis results.**

| Model | ${m\mathrm{AP}}_{kpt}$/% | F1/% | FPS/(frames/s) |
| --- | --- | --- | --- |
| YOLOv8-Pose | 92.10±0.38 | 89.48±0.89 | 145.5±2.87 |
| SRD-YOLO | 96.37±0.26 | 94.40±0.31 | 166.83±2.77 |
| P (Significance Test) | $3.64\times{10}^{-6}<0.01$ | ${5.71\times10}^{-5}<0.01$ | ${1.16\times10}^{-5}<0.01$ |
